# Impact of Cooking Procedures on Coccidiostats in Poultry Muscle

**DOI:** 10.3390/antibiotics14060586

**Published:** 2025-06-07

**Authors:** Rui R. Martins, André M. P. T. Pereira, Liliana J. G. Silva, Sofia C. Duarte, Andreia Freitas, Angelina Pena

**Affiliations:** 1 LAQV, REQUIMTE, Laboratory of Bromatology and Pharmacognosy, Faculty of Pharmacy, University of Coimbra, Polo III, Azinhaga de Santa Comba, 3000-548 Coimbra, Portugal; martins.rio.rui@gmail.com (R.R.M.); ljgsilva@ff.uc.pt (L.J.G.S.); sofia.duarte@euvg.pt (S.C.D.); apena@ci.uc.pt (A.P.); 2 Centre of Studies in Animal and Veterinary Science (CECAV), University of Trás-os Montes e Alto Douro (UTAD), Apartado 1013, 5001-801 Vila Real, Portugal; 3 Centro de Investigação Vasco da Gama, Escola Universitária Vasco da Gama (EUVG), Av. José R. Sousa Fernandes 197, Campus Universitário de Lordemão, 3020-210 Coimbra, Portugal; 4 National Institute for Agricultural and Veterinary Research (INIAV), I.P., Av. da República, Quinta do Marquês, 2780-157 Oeiras, Portugal; andreia.freitas@iniav.pt; 5 Associated Laboratory for Green Chemistry of the Network of Chemistry and Technology, REQUIMTE/LAQV, R. D. Manuel II, Apartado 55142, 4051-401 Porto, Portugal

**Keywords:** poultry meat, antimicrobials, food contaminants, cooking methods, coccidiostat residues, UHPLC-MS/MS

## Abstract

**Background/Objectives:** Poultry meat is a popular and nutritious food, valued for its high protein content and healthy fat profile. However, like other animal products, it can contain pharmaceutical residues, including coccidiostats, antimicrobials commonly used to prevent parasitic infections caused by Eimeria species. While most monitoring focuses on raw meat, it is important to understand how these compounds behave during cooking to assess potential health risks better and ensure food safety. **Methods:** This study examined how five different cooking methods (roasting, grilling, and microwaving, beer and wine marinating) affect the levels of eight coccidiostat residues in 45 samples of poultry muscle collected from a supermarket located in the center of mainland Portugal from May to July 2024. After applying different cooking procedures, ionophore and synthetic coccidiostat residue levels were measured using solid–liquid extraction followed by ultrahigh-performance liquid chromatography with tandem mass spectrometry (UHPLC-MS/MS). Results are expressed as percentages of the original concentrations: 100% indicates stability, values above 100% suggest a relative increase (often due to moisture loss), and values below 100% reflect a decrease, likely from heat degradation. **Results:** Roasting, grilling, and microwaving all increased residue concentrations—up to 198.5%, 180.1%, and 158.4%, respectively. In contrast, marinating meat in wine or beer before cooking reduced residues to 73.1% and 72.0%, suggesting a mitigating effect. The initial concentration also influenced the outcome: samples fortified at the maximum residue limit (MRL) had an overall higher mean concentration after cooking (148.3%,) than those fortified at twice the MRL (2 MRL), which averaged 124.5%. **Conclusions:** These results show that cooking can significantly alter coccidiostat residue levels depending on the cooking procedures and initial concentration. Ongoing monitoring and further research are essential to better understand how cooking affects these residues and their by-products. This knowledge is key to improving food safety practices and refining consumer health risk assessments.

## 1. Introduction

The poultry sector plays a vital role in meeting the growing global demand for meat and eggs, driven by population growth in emerging nations. It supplies more than one third of the protein essential for human nutrition [1].

In addition to its high protein content, poultry meat is a valuable supply of phosphorus, as well as other essential minerals and B-complex vitamins. It has a lower fat content compared to the majority of beef and pork cuts and is also low in unhealthy trans fats. Moreover, it is rich in beneficial monounsaturated fats, which account for around half of its total fat content. Globally, chicken is the most consumed meat due to its affordability, low fat content, and few religious and cultural restrictions. Consequently, poultry production is steadily increasing and is expected to continue growing in the future to meet rising global demand [2].

Optimal intestinal health is crucial for the efficient digestion, absorption, and metabolism of developing avian species. Coccidiosis is a highly contagious infectious disease caused by parasitic single-celled protozoa of the genus *Eimeria*, specifically belonging to the coccidia subclass. This disease leads to weight loss, impaired feed conversion, and decreased egg production [3,4]. The lower meat yield and quality, along with increased susceptibility to diseases and high mortality rates in its acute form, result in significant economic losses, amounting to up to USD 3 billion yearly [5].

Out of the over 600 known species of coccidia, only nine species of *Eimeria* have been found in chickens. The most virulent of these include *E. tenella*, *E. necatrix*, *E. maxima*, *E. acervulina*, *E. mitis*, *E. praecox*, and *E. brunetti* [6,7]. One of the most efficient means of a coccidiosis control strategy involves the use of antiprotozoal agents and coccidiostats as additives in feed mixtures. Together with the implementation of biosecurity measures and environmental hygiene practices, these strategies aim to ensure the long-term viability of chicken production [8].

At present, there are 11 coccidiostats approved as feed additives in Europe for the prevention of infections in broilers. Currently, they are classified into two groups, namely ionophores, produced by fermentation of *Streptomyces* spp. and *Actinomadura* spp. (monensin, salinomycin, narasin, lasalocid, semduramicin, and maduramicin) and the chemical or “synthetic” coccidiostats (decoquinate, diclazuril, halofuginone, nicarbazin, robenidine, and amprolium) [9]. Ionophore coccidiostats are more widely used than synthetic alternatives due to their efficacy, safety profile, cost-effectiveness, lower resistance risk, and regulatory acceptance. These factors make them the preferred option for managing coccidiosis in livestock and poultry, particularly in large-scale commercial operations [10]. However, owing to their natural origin and inherent antimicrobial properties, they are categorized as antibiotics. While primarily employed for the prophylactic and therapeutic management of coccidiosis, their application raises significant concerns regarding the potential emergence of antimicrobial resistance. This is not limited to *Eimeria* spp., but extends to bacterial populations, particularly Gram-positive organisms such as Clostridium perfringens, due to their broad-spectrum antimicrobial activity [11].

Ionophores exert selective pressure on diverse microbial populations, notably targeting protozoan parasites such as *Eimeria* spp. while also affecting the gut microbiota due to their broad-spectrum activity. This dual action may contribute to the emergence of resistance through cross-resistance mechanisms or co-selection of antimicrobial-resistance genes within bacterial communities [12]. Additionally, ionophores exhibit environmental persistence: residues present in poultry litter and manure can remain active in soil and aquatic environments, thereby extending their antimicrobial influence beyond the host. Although ionophores are not employed in human medicine, their use in animal production raises concerns regarding the co-selection of resistance genes that are genetically linked to those conferring resistance to medically important antibiotics, potentially facilitating their dissemination through horizontal gene transfer [13].

Unlike ionophores, synthetic coccidiostats generally lack antibacterial activity and do not significantly affect bacterial populations [14]. Although vaccination against coccidiosis is available, its high cost, limited effectiveness, and strain-specific immunity make it less viable for widespread application in poultry production. Unlike coccidiostats, which provide immediate control of infections, vaccines require time to induce immunity, leaving birds susceptible to early-stage infections. As a result, coccidiostats remain the primary therapeutic tool for managing coccidiosis in poultry farming [15]. However, the extensive and prolonged use of coccidiostats raises concerns about drug resistance. Continuous exposure, limited rotation of active compounds, and cross-resistance among Eimeria strains contribute to a decline in drug efficacy over time. Although rotation and shuttle programs can slow resistance development, they are not definitive solutions [16].

Regarding the health risks to humans following exposure to high levels of these drug residues in the edible parts of animals, it is important to bear in mind that most coccidiostats are lipophilic (Table S1, Supplementary Material). This means that their residues tend to accumulate in tissues with higher fat content, such as fat, skin, and liver, which can pose health hazards. To mitigate these risks, it is crucial to avoid using higher doses than those recommended, adhere to proper withdrawal periods, and ensure that good veterinary practices are followed [17]. In fact, the use of these substances in laying hens is prohibited because it could expose consumers to these lipophilic and harmful compounds in eggs [18].

Pharmacokinetic studies of these drugs in different animal species intended for human consumption are crucial for understanding their availability, absorption, metabolism, and elimination, including both the original drug and any active by-products. These studies are required to determine appropriate withdrawal periods for specific coccidiostats in various edible tissues. Establishing precise safety intervals is essential to ensure that these drug residues decline to acceptable levels before slaughter, minimizing consumer exposure. Given the variability in drug metabolism and tissue distribution, tailored withdrawal times for different tissues, such as muscle, liver, and fat, are necessary to enhance food safety and regulatory compliance [19].

The European Food Safety Authority (EFSA) and the Joint Food and Agriculture Organization of the United Nations–World Health Organization Expert Committee on Food Additives (JECFA), established acceptable daily intakes (ADIs) for all authorized coccidiostats of between 0.03 µg kg^−1^ of body weight (bw) per day for halofuginone (Agency and Medicines, 2013) and 200 µg kg^−1^ bw per day for nicarbazin [20].

Maximum residue limits (MRLs) in animal tissues serve as a risk management measure to guarantee consumer safety and promote global trade. Ensuring that the consumption of veterinary drug residues remains at or below the MRL concentrations guarantees that eventual human exposure will not surpass the defined levels of ADI [21]. In the European Union (EU), MRLs are regulated by Regulation (EC) No. 470/2009 [22] and established by the European Medicines Agency (EMA). The specific MRLs in raw poultry muscle vary from 8 µg kg^−1^ (monensin) to 4000 µg kg^−1^ (dinitrocarbanilide, nicarbazin residue marker) (Table S2, Supplementary Material). These values are subject to periodic updates based on risk assessments conducted by the EFSA.

Current MRLs were established on raw poultry tissues, but most chicken meat is consumed after proper cooking procedures (thermal or taste-enhancement treatments or both), which effectively eliminate pathogenic microorganisms, ensuring its safety for consumption and for the purpose of enhancing taste, aroma, and shelf life.

Therefore, this work aimed to determine the influence that different cooking procedures have on the concentration of coccidiostats in poultry muscle to evaluate real consumers’ exposure to coccidiostat residues and their influence on food safety and human health. After applying different cooking procedures, roasting, grilling, microwaving, and marinating (beer and wine), ionophore and synthetic coccidiostat residue levels were measured using solid–liquid extraction followed by ultrahigh-performance liquid chromatography with quadrupole tandem mass spectrometry (UHPLC-MS/MS) to evaluate their effect on the presence of the compounds in poultry meat.

## 2. Results

### 2.1. Method Validation

The analytical method has previously been validated, including key parameters such as linearity, matrix effect (ME), limit of detection (LOD), limits of quantification (LOQ), accuracy, and precision [23]. Nonetheless, revalidation was performed, and LOD and LOQ are presented in Table S3 (Supplementary Material).

### 2.2. Comparison Between Coccidiostats and Cooking Processes

The results presented in this study are reported as a percentage of the concentration in the processed sample relative to the initial concentration, without considering weight loss during preparation (concentration variation). Results are expressed as percentages of the original concentrations: 100% indicates stability, values above 100% suggest a relative increase, and values below 100% reflect a decrease.

#### 2.2.1. Comparison Between Groups of Coccidiostats

**Figure 1:**
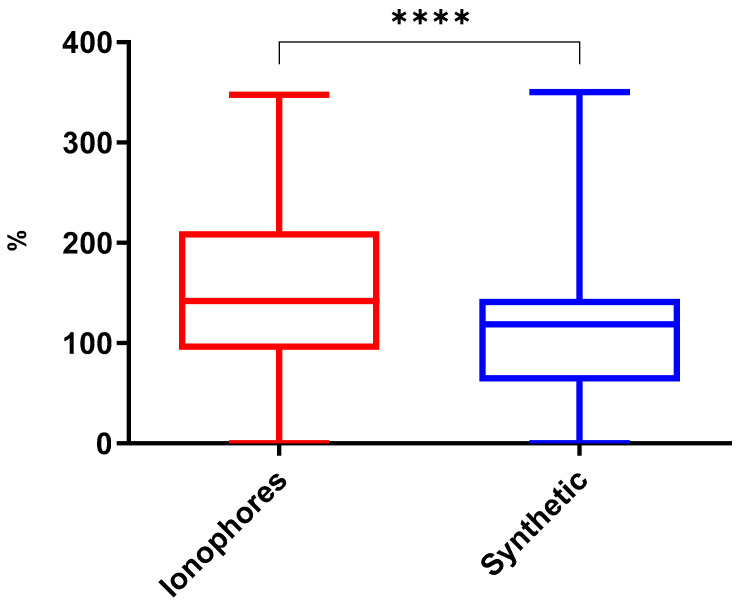
Box-and-whisker plots for the concentrations of coccidiostats after various cooking methods. The results presented in this study are reported as a percentage of the concentration in the processed sample relative to the initial concentration. Asterisks indicate statistical significance: **** *p* < 0.0001.

The coccidiostat groups, pharmacologically active compounds added to animal feed or water to inhibit the growth and reproduction of Eimeria parasites in the intestines of food-producing animals present in this study, ionophores and synthetic, were submitted the various culinary methods (roast, microwave, grill, beer marinade, and wine marinade). Ionophore coccidiostats presented a mean of 151.3%, with a 95% confidence interval (CI) between 139.1% and 163.5%, while the synthetic group had a lower mean, 111.6%, with a 95% CI between 99.5.8% and 123.6%. There was a statistically significant difference between them (*p* ˂ 0.0001) (Figure 1).

The results presented in this study are reported as a percentage of the concentration in the processed samples relative to the initial concentration. Asterisks indicate statistical significance: **** *p* < 0.0001, *** *p* = 0.0001 to 0.001, ** *p* = 0.001 to 0.01, * *p* = 0.01 to 0.05.

Ionophore and synthetic coccidiostats behave differently during cooking due to their distinct chemical structures, stability, and solubility properties. Ionophore coccidiostats tend to be more thermally stable due to their polyether structures, which provide resistance to heat. Their lipophilic nature causes them to concentrate in fat-rich areas, potentially affecting their degradation or redistribution during cooking [24]. These results are in line with these antibiotics’ environmental persistence. This behavior is consistent with findings from previous studies [25,26], which reported persistence of ionophores such as monensin and salinomycin after cooking, often exceeding initial levels due to weight loss and fat affinity.

In contrast, synthetic coccidiostats are generally less heat-stable, breaking down more easily at high temperatures. Being more hydrophilic, they dissolve more readily in cooking juices, potentially leading to greater reduction during food preparation. These results are consistent with prior studies that observed significant reductions in residues of synthetic coccidiostats like nicarbazin, diclazuril, and robenidine during boiling or stewing [15,27].

Additionally, cooking methods such as roasting and grilling, which result in significant moisture loss, can lead to an apparent concentration of residues due to the reduction in water content [28]. These changes in coccidiostat residue levels are critical to food safety evaluations, as they directly affect the actual exposure of consumers to these compounds in the final cooked product [8]. They also underscore the need to evaluate not just the parent compound levels but also degradation products when assessing consumer exposure and food safety. Although the proposed approach using spiked poultry meat seemed the best approach to this issue, spiking meat has limitations, since it does not mimic real-life residue distribution, metabolism, or tissue binding.

#### 2.2.2. Comparison Between Coccidiostats

**Figure 2:**
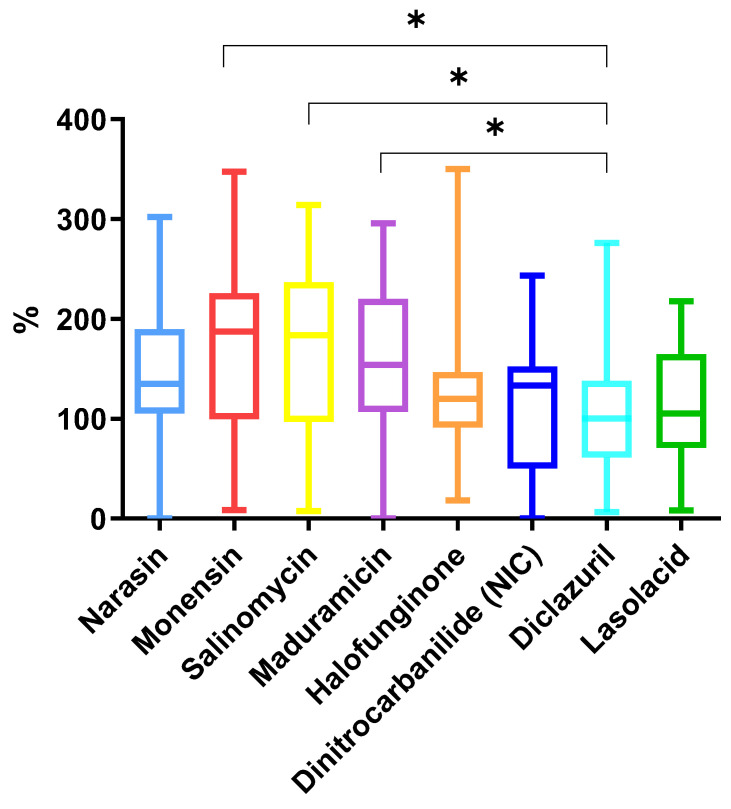
Box-and-whisker plots for the concentrations of the different coccidiostats after cooking. The results presented in this study are reported as a percentage of the concentration in the processed sample relative to the initial concentration. Asterisks indicate statistical significance: * *p* = 0.01 to 0.05.

After the cooking procedures, the compounds presented the following average concentration percentage values: salinomycin 170.2%; monensin 171.4%; maduramicin 160.1%; narasin 141.4%; halofuginone 120.2%; dinitrocarbanilide (nicarbazin residue marker) 111.8%; and diclazuril 102.7% (Figure 2). Monensin, salinomycin and maduramicin presented statistically significant differences with regard to their final concentrations with the compound diclazuril.

The results presented in this study are reported as a percentage of the concentration in the processed samples relative to the initial concentration. Asterisks indicate statistical significance: **** *p* < 0.0001, *** *p* = 0.0001 to 0.001, ** *p* = 0.001 to 0.01, * *p* = 0.01 to 0.05.

In line with the coccidiostat group characteristics, ionophore coccidiostats like monensin, salinomycin, and maduramicin are more heat-stable and therefore tend to remain in higher concentrations after dry-heat cooking methods. The breakdown of coccidiostats is particularly evident in synthetic coccidiostats, such as diclazuril and dinitrocarbanilide (nicarbazin residue market), which are more susceptible to acid-induced degradation [29,30]. These findings are consistent with previous studies demonstrating that polyether ionophores are resistant to thermal degradation and tend to accumulate in fat matrices during roasting or grilling [25].

In contrast, the more hydrophilic and thermolabile nature of synthetic coccidiostats contributes to their partial loss during cooking, particularly under moist conditions. For example, dinitrocarbanilide has been shown to degrade under heat, and the formation of p-nitroaniline as a thermal breakdown product during boiling and grilling has been identified [27,31,32]. Other authors also detected the formation of p-nitroaniline as a degradation product of dinitrocarbanilide in chicken breast subjected to heat treatment. Their findings demonstrated that thermal processing not only affects the parent compound concentration but may also generate secondary compounds of toxicological relevance [8].

The relatively lower concentration values observed for diclazuril and dinitrocarbanilide in this study align with these prior reports, confirming that synthetic coccidiostats are more likely to undergo transformation or migrate into cooking fluids.

#### 2.2.3. Comparison Between Cooking Methods

**Figure 3:**
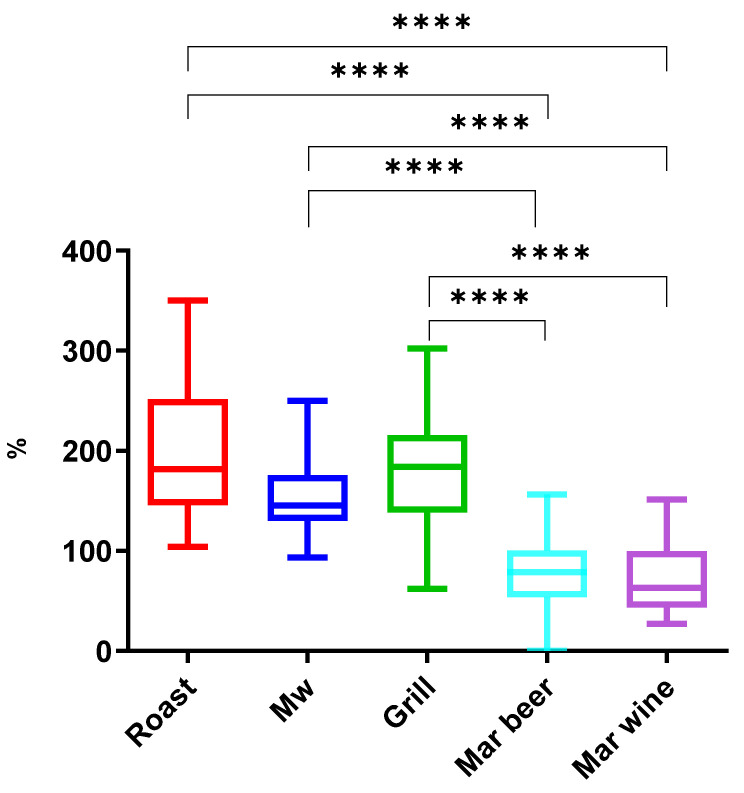
Box-and-whisker plots comparing the influence of different cooking methods in coccidiostat concentrations. The results presented in this study are reported as a percentage of the concentration in the processed sample relative to the initial concentration. Asterisks indicate statistical significance: **** *p* < 0.0001.

The roast, grill, and microwave culinary methods presented average initial concentrations of 198.5%, 180.1%, and 158.4%, respectively. On the other hand, the beer marinade and wine marinade processes showed a reduction in relation to the initial content, with average values of 73.1% and 72.0%, respectively (Figure 3). Thus, a statistically significant difference was found between the roast, grill, and microwave methods and the marinades (wine and beer). In the case of marinades, it is important to note that the removal values found were more homogeneous and uniform than in the other culinary methods used.

Different cooking methods presented varying effects on the concentration of coccidiostat residues in poultry muscle due to differences in heat exposure, moisture loss, and chemical interactions. Microwaving, roasting, and grilling tend to increase the apparent concentration of coccidiostats. This is primarily due to moisture loss, which concentrates the residues in the remaining tissue, even if some thermal degradation occurs. Microwave cooking leads to a rapid reduction in water content, while roasting and grilling expose the meat to prolonged or direct dry heat, further enhancing residue concentration.

In contrast, beer and wine marination contribute to a reduction in coccidiostat levels. The acidic pH and ethanol content in these marinades likely promote chemical degradation or leaching of residues into the liquid [33].

Overall, dry-heat methods (microwave, roasting, and grilling) tend to increase apparent residue levels, while marination in beer or wine helps to reduce coccidiostat concentrations, likely due to solvent effects and pH-related degradation.

These findings suggest that moist-heat and acidified preparations are more effective at reducing consumer exposure to certain synthetic coccidiostats, while dry-heat methods may inadvertently concentrate lipophilic residues, especially if initial levels are high.

#### 2.2.4. Comparison Between Maximum Residue Level and Twice-Maximum-Residue Level Concentrations

**Figure 4:**
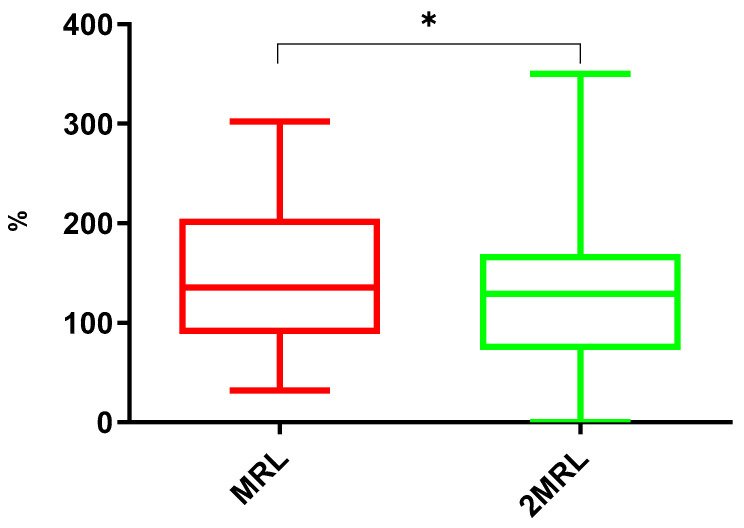
Box-and-whisker plots for the comparison between the two groups analyzed. The results presented in this study are reported as a percentage of the concentration in the processed sample relative to the initial concentration. Asterisks indicate statistical significance: * *p* = 0.01 to 0.05.

The results indicated statistically significant differences between the two groups analyzed (*p* = 0.0198) (Figure 4). The MRL group had a mean of 148.3%, with a 95% CI between 135.5% and 161.1%. The 2 MRL group presented a lower mean of 124.5%, with a 95% CI between 111.5% and 137.4%. From the obtained results, it was possible to conclude that across all cooking methods, at the highest concentration (2 MRL), the removal of compounds after preparation was more efficient than in samples with lower coccidiostat concentrations (MRL).

This trend can be justified based on several key factors. In the case of marination, at higher fortification levels (2 MRL), there is a greater concentration gradient between the meat and the marinade liquid. This leads to more efficient leaching of residues into the marinade, particularly for synthetic coccidiostats, which are more soluble in acidic and ethanol-rich environments.

Regarding the other cooking procedures, another factor that explains this pattern is the enhanced thermal degradation. Many coccidiostats degrade when exposed to heat. At 2 MRL, the absolute quantity of residues available for breakdown is higher, leading to a greater total reduction after cooking. Dry-heat methods (microwave, roasting, grilling) concentrate residues through moisture loss, but they also promote some thermal degradation, especially at higher initial concentrations.

The evaporation and loss during cooking also impacts the coccidiostat concentration. Some coccidiostats, like narasin and dinitrocarbanilide, are partially volatile or break down into by-products at high temperatures [34]. At 2 MRL, more molecules are available for volatilization, leading to a higher percentage of loss compared to MRL samples.

Finally, the binding site saturation in muscle tissue can also influence the results. At MRL, coccidiostats are more tightly bounded to proteins and lipids in muscle. At 2 MRL, the binding capacity of the meat matrix may become saturated, leaving more free residues that are easier to extract, degrade, or evaporate [26].

## 3. Materials and Methods

### 3.1. Sampling and Cooking Methods

A total of 45 raw whole samples of the same brand and batch were collected from a supermarket located in the center of mainland Portugal from May to July 2024.

Samples were identified and characterized by sampling date, location, and producer. Then, muscle samples were removed and frozen at −18 °C or lower until processing.

The microwaving process was performed using a household turntable microwave oven. The meat pieces were cooked at full power (700 W, 2450 MHz) for 5 min to ensure the food reached a safe internal temperature (74 °C), and then allowed to cool naturally to room temperature (22 °C± 2 °C).

In the roasting process, meat samples were subjected to oven baking in a preheated convection oven at 180 °C for 30 min. The samples were placed on a heat-resistant tray and baked without additional fat or seasoning. After baking, the samples were removed from the oven and allowed to cool naturally to room temperature (22 ± 2 °C) before further analysis.

Grilling was performed on a preheated electric grill at 230 °C. The samples were cooked for 6 min per side, totaling 12 min, ensuring an internal temperature of at least 75 °C. The grill plates ensured direct heat contact, mimicking real-life grilling conditions. In the post-cooking processing, samples were cooled naturally to room temperature (22 ± 2 °C) before further analysis.

For beer marination, meat samples were submerged in a beer (pilsner) solution at a 1:1 ratio (*w*/*v*) and aromatic parts of herbs and spices such as thyme, garlic, and parsley in sterile glass containers. The marination process was carried out at 4 °C for 12 h under constant refrigeration. After marination, the samples were drained, excess liquid was removed, and the meat was left at room temperature (22 ± 2 °C) before further processing or analysis.

The wine marination process followed the same protocol as beer marination. Meat samples were immersed in a wine solution at a 1:1 ratio (*w*/*v*) and aromatic parts of herbs and spices such as thyme, garlic, and parsley and stored at 4 °C for 12 h. The acidity and polyphenols in Portuguese wines contribute to both flavor and potential chemical changes in the meat. After marination, the samples were drained, excess liquid was removed, and the meat was equilibrated to room temperature (22 ± 2 °C) before subsequent processing or analysis.

All assays were conducted in triplicate. Blank samples were used for each cooking procedure (n = 15), and fortifications were performed at the MRL (n = 15) and at double MRL (2 MRL) (n = 15) for each coccidiostat and for each cooking procedure.

### 3.2. Chemicals, Reagents, and Standard Solutions

Certified reference standards of coccidiostats—sodium lasalocid, sodium narasin, sodium salinomycin, sodium monensin, sodium maduramicin, halofuginone, diclazuril, and 4,4′-dinitrocarbanilide (residue marker of nicarbazin)—of ≥98% purity were obtained from Sigma Chemical Co. (St. Louis, MO, USA). Acetonitrile was also acquired from Sigma, while formic acid and dimethyl sulfoxide (DMSO) were purchased from Merck (Darmstadt, Germany). Milli-Q bi-distilled water was produced daily using a purification system (Millipore, Bedford, MA, USA).

Stock standard solutions were prepared at 1 mg mL^−1^ in acetonitrile. Calibration standards were prepared both in solvent (mobile phase A) and in matrix, based on the MRLs established for each analyte. Matrix-matched calibration curves were prepared by spiking blank poultry meat at five levels: blank, 0.5 MRL, 1 MRL, 1.5 MRL, and 2 MRL. A working internal standard solution containing 1 µg·mL^−1^ of nigericin and dinitrocarbanilide-d_8_ was prepared through appropriate dilutions. All standard and working solutions were stored at −25 °C for up to 12 months.

### 3.3. Sample Extraction

Sample extraction and chromatography analysis were performed based on a previous study [23]. Approximately 3 g of minced and homogenized meat was extracted with 10 mL of acetonitrile using vortex mixing, ultrasound treatment, and vertical shaking. After centrifugation (5444× *g*, 15 min, 4 °C), the supernatant was evaporated to dryness at 45 °C under nitrogen. The residue was redissolved in mobile phase A, microfiltered, and analyzed by ultrahigh-performance liquid chromatography–MS/MS (UHPLC-MS/MS).

### 3.4. UHPLC-MS/MS Analysis

Quantification of coccidiostats was performed using a UHPLC system (Nexera X2, Shimadzu, Kyoto, Japan) coupled with a QTRAP 5500+ triple quadrupole mass spectrometer (Sciex, Foster City, CA, USA). Chromatographic separation was achieved using a Phenomenex Kinetex biphenyl column (2.1 × 50 mm, 1.7 µm, 100 Å), maintained at 40 °C. The injection volume was 10 µL, and the flow rate was 500 µL min^−1^.

The mobile phase consisted of 0.1% formic acid in water (A) and acetonitrile (B). The gradient program was as follows: 0–6 min, linear from 100% A to 100% B; held at 100% B until 9 min; then returned to 100% A by 10 min, for a total run time of 11 min.

Mass spectrometric detection was carried out using an electrospray ionization (ESI) source in both positive and negative ionization modes, operating at 500 °C. Data acquisition was conducted in multiple reaction monitoring (MRM) mode using Analyst TF 1.8.1 software, and peak integration was performed with MultiQuant (Sciex).

The determination of both LOD and LOQ was based on the ICH guidelines by multiplying the standard deviation associated with the 20 blank samples analyzed by 3.3 (LOD) or 10 (LOQ) and dividing this value by the slope of the calibration curve. The analysis of those 20 blanks was also used to evaluate the selectivity and specificity. The chromatograms of all compounds in the blank samples were found to be free of any interference in the expected retention time of the targeted coccidiostats.

Linearity was evaluated using standards and matrix-matched calibration curves with concentration levels equivalent to 0.5 MRL, MRL, 1.5 MRL and 2 MRL.

### 3.5. Statistical Analysis

All statistical analyses were performed using GraphPad Prism v6.01 (GraphPad Software, San Diego, CA, USA). Normality of data distribution was assessed using the D’Agostino–Pearson test. Given that most datasets did not meet assumptions of normality or equal variance, non-parametric methods were employed. The Kruskal–Wallis test with Dunn’s post hoc multiple comparison was applied to detect statistically significant differences among groups.

For concentrations below the limit of detection (LOD) and limit of quantification (LOQ), values were estimated as ½ LOD and ½ LOQ, respectively, for mean calculations. A significance threshold of *p* < 0.05 was applied. Statistical differences are indicated as follows:

**** *p* < 0.0001, *** *p* = 0.0001–0.001, ** *p* = 0.001–0.01, * *p* = 0.01–0.05.

## 4. Conclusions

This study highlights the complex influence of cooking methods on the behavior of coccidiostat residues in poultry meat. The extent of residue reduction or concentration varies depending on the type of coccidiostat, ionophore or synthetic, as well as the cooking technique applied. Ionophore coccidiostats (antibiotics), due to their thermal stability and lipophilicity, tend to persist at higher levels following dry-heat cooking methods, whereas synthetic coccidiostats, which are more hydrophilic and thermolabile, are more susceptible to degradation, particularly under moist-heat conditions such as beer or wine marination.

At higher initial fortification levels (2 MRL), residue removal was more pronounced, likely due to enhanced mass transfer, thermal degradation, and solvent interactions during cooking. Among all methods evaluated, marination emerged as the most effective in reducing coccidiostat levels, especially at elevated concentrations (2 MRL). However, despite observable reductions, cooking alone cannot be considered a reliable strategy to ensure that residue levels consistently fall below established safety thresholds. Moreover, recent evidence suggests that thermal degradation of certain synthetic coccidiostats, such as 4,4′-dinitrocarbanilide, may lead to the formation of potentially hazardous metabolites, including p-nitroaniline, a compound with recognized toxicological relevance. These findings indicate that cooking can not only reduce parent compound residues but also generate new compounds of concern, thereby complicating the overall food safety profile.

Therefore, from a food safety and toxicological standpoint, regulatory compliance must be achieved prior to food preparation, as thermal processing cannot be solely relied upon to mitigate the risks associated with coccidiostat residues in poultry meat.

## Data Availability

The original contributions presented in this study are included in the article/Supplementary Materials. Further inquiries can be directed to the corresponding author.

## Supplementary Materials

The following supporting information can be downloaded at https://www.mdpi.com/article/10.3390/antibiotics14060586/s1. Table S1: Physicochemical characteristics of coccidiostats; Table S2: Maximum residue levels of coccidiostats; Table S3: Limits of detection (LOD) and limits of quantification (LOQ) of the analytical methodology.
