# Supplementary material for: Impact of Cooking Procedures on Coccidiostats in Poultry Muscle
Source: Antibiotics (Basel). 2025 Jun 7;14(6):586. doi: 10.3390/antibiotics14060586 (PMC12189227; doi:10.3390/antibiotics14060586)
Supplement: Supplementary file 1 [file antibiotics-14-00586-s001.zip › antibiotics-3667317-supplementary.pdf]

Supplementary Material for:

Impact of cooking procedures on coccidiostats in poultry muscle

Rui R. Martins [1,2,3], André M. P. T. Pereira [1,*], Liliana J. G. Silva [1], Sofia C. Duarte [1,3], Andreia Freitas [4,5] and Angelina Pena [1]

[1] LAQV, REQUIMTE, Laboratory of Bromatology and Pharmacognosy, Faculty of Pharmacy, University of Coimbra, Polo III, Azinhaga de Santa Comba, 3000-548 Coimbra, Portugal
[2] Centre of Studies in Animal and Veterinary Science (CECAV), University of Trás-os Montes e Alto Douro (UTAD), Apartado 1013, 5001-801 Vila Real, Portugal
[3] Centro de Investigação Vasco da Gama, Escola Universitária Vasco da Gama (EUVG), Av. José R. Sousa Fernandes 197, Campus Universitário de Lordemão, 3020-210 Coimbra, Portugal
[4] National Institute for Agricultural and Veterinary Research (INIAV), I.P., Av. da República, Quinta do Marquês, 2780-157 Oeiras, Portugal
[5] Associated Laboratory for Green Chemistry of the Network of Chemistry and Technology, REQUIMTE/LAQV, R. D. Manuel II, Apartado 55142, 4051-401 Porto, Portugal
* Correspondence: e-mail@e-mail.com; Tel.: (optional; include country code; if there are multiple corresponding authors, add author initials)

Table S1. Physicochemical characteristics of coccidiostats.

Compound	Type	Chemical Class	Water Solubility	Lipophilicity (log P)	Stability
**Lasalocid**	Ionophore	Polyether carboxylic acid	Very low	~5.9	Degrades with heat & light
**Narasin**	Ionophore	Polyether antibiotic	Very low	~6.4	Stable in feed, light-sensitive
**Salinomycin**	Ionophore	Polyether antibiotic	Very low	~8.6	Heat- and light-sensitive
**Monensin**	Ionophore	Polyether carboxylic acid	Very low	~5.0	Stable in dry, cool storage
**Maduramicin**	Ionophore	Polyether antibiotic	Insoluble	~6.5	Light-sensitive, moderately stable
**Halofuginone**	Non-ionophore	Quinazolinone alkaloid	Slightly soluble	~1.3	Stable in feed
**Robenidine**	Non-ionophore	Guanidine derivative	Poorly soluble	~2.9	Sensitive to acidic pH
**Diclazuril**	Non-ionophore	Benzeneacetonitrile	Practically insoluble	~2.6	Light-sensitive, stable dry
**Decoquinate**	Non-ionophore	Quinolone derivative	Insoluble	~5.2	Stable in feed

Table S2. Maximum residue levels of coccidiostats.

Generic Name/Applicable Legislation	Target Species	LMR
**Monensin** (Commission Regulation No. 1096/2008)	Broiler	25 µg/kg of skin/ fat;
	Turkeys (max. 16 weeks)	8 µg/kg of liver, kidney and muscle.<br>2 µg/kg of skin /fat, kidney and muscle;
	Laying hens	8 µg/kg of liver.
**Salinomicin** (Commission Regulation No. 496/2007)	Broiler	150 µg/kg of liver;<br>40 µg/kg of kidney;<br>15 µg/kg of muscle,<br>150 µg/kg of skin/fat.
	Laying hens	5 µg/kg of liver; 2 µg/kg of kidney;<br>2 µg/kg of muscle, 2 µg/kg of skin/fat.
	Eggs	3 µg/kg
**Narasin** (Commission Regulation No. 885/2010)	Broiler	50 µg/kg all edible tissues
	Eggs	2 µg/kg
**Lasalocid** (Commission Regulation No. 37/2010)	Broiler	20 µg/kg of muscle;<br>100 µg/kg of skin/fat, liver;<br>50 µg/kg of kidney
	Turkey	
	Laying hens	-
	Pheasants, guinea fowl, quails and partridges, other than poultry.	-
	Eggs	5 µg/kg
**Maduramicin** (Commission Regulation No. 388/2011)	Broiler	150 µg/kg of liver, skin/fat;<br>100 µg/kg of kidney;<br>30 µg/kg of muscle
	Turkey (max. 16 weeks)	-
	Eggs	12 µg/kg
**Robenidine** (Commission Regulation No. 124/2009 and Community register of feed additives)	Broiler	800 µg/kg of liver;<br>350 µg/kg of kidney;<br>200 µg/kg of muscle;<br>1 300 µg/kg of skin/fat
	Turkeys	400 µg/kg of skin/fat<br>400 µg/kg of liver<br>200 µg kg of kidney<br>200 µg/kg of muscle
	Eggs	25 µg/kg
**Halofuginone** (Commission Regulation No. 37/2010 and Commission Regulation No. 124/2009)	Broiler	-
	Bovine	10 µg/kg
	Eggs	6 µg/kg
**Diclazuril** (Commission Regulation No. 971 and 976/2008)	Broiler	1500 µg/kg of liver;<br>1000 µg/kg of kidney;<br>500 µg/kg of muscle;
	Turkeys	500 µg/kg of skin/fat.

	Laying hens	-
	Eggs	2 µg/kg
**Decoquinate**<br>(Commission Regulation No. 37/2010)	Broiler	1000 µg/kg liver and skin/fat;<br>800 µg/kg of kidney;<br>500 µg/kg of muscle
**Nicarbazin**<br>(Commission Regulation No. 124/2009)	Broiler	15,000 µg de DNC/kg of liver;<br>6000 µg de DNC/kg of kidney;<br>4000 µg de DNC/kg of muscle and skin/fat
	Eggs	300 µg/kg

Table S3. Limits of detection (LOQs) and limits of quantification (LOQs) of the analytical methodology.

**Coccidiostat**	**LOD**	**LOQ**
Lasalocid	0.9	1.16
Narasin	0.21	0.63
Monensin	0.038	0.26
Salinomycin	0.037	0.11
Maduramycin	0.029	0.087
Halofuginone	0.29	0.89
Dinitrocarbanilide	8.13	27.47
Diclazuril	11.46	34.83
